# Creating a human-induced pluripotent stem cell-based NKX2.5 reporter gene assay for developmental toxicity testing

**DOI:** 10.1007/s00204-021-03018-y

**Published:** 2021-03-04

**Authors:** Karin Lauschke, Andreas Frederik Treschow, Mikkel Aabech Rasmussen, Nichlas Davidsen, Bjørn Holst, Jenny Emnéus, Camilla Taxvig, Anne Marie Vinggaard

**Affiliations:** 1 National Food Institute, Technical University of Denmark, Kemitorvet, Building 201, 2800 Kongens Lyngby, Denmark; 2 Department for Biotechnology and Biomedicine, Technical University of Denmark, Kongens Lyngby, Denmark; 3 Bioneer A/S, Hørsholm, Denmark

**Keywords:** CRISPR/Cas9, Human- induced pluripotent stem cell, Thalidomide, Luminescence, Reporter cell line assay

## Abstract

To test large numbers of chemicals for developmental toxicity, rapid in vitro tests with standardized readouts for automated data acquisition are needed. However, the most widely used assay, the embryonic stem cell test, relies on the counting of beating embryoid bodies by visual inspection, which is laborious and time consuming. We previously developed the PluriBeat assay based on differentiation of human induced pluripotent stem cells (hiPSC) that we demonstrated to be predictive for known teratogens at relevant concentrations using the readout of beating cardiomyocytes. Here, we report the development of a novel assay, which we term the PluriLum assay, where we have introduced a luciferase reporter gene into the locus of *NKX2.5* of our hiPSC line. This enabled us to measure luminescence intensities instead of counting beating cardiomyocytes, which is less labor intensive. We established two *NKX2.5* reporter cell lines and validated their pluripotency and genetic stability. Moreover, we confirmed that the genetically engineered *NKX2.5* reporter cell line differentiated into cardiomyocytes with the same efficiency as the original wild-type line. We then exposed the cells to valproic acid (25–300 μM) and thalidomide (0.1–36 µM) and compared the PluriBeat readout of the cardiomyocytes with the luminescence intensity of the PluriLum assay. The results showed that thalidomide decreased luminescence intensity significantly with a higher potency and efficacy compared to the beating readout. With this, we have developed a novel hiPSC-based assay with a standardized readout that may have the potential for higher throughput screening for developmental toxicity.

**Supplementary Information:**

The online version contains supplementary material available at 10.1007/s00204-021-03018-y.

## Introduction

Safeguarding developing human embryos and fetuses is very important as pregnant women are exposed to an increasing number of chemicals that are potentially hazardous at human exposure levels (Worley et al. 2018). However, testing for developmental toxicity is one of the most challenging areas of toxicology, because the required animal experiments are costly, labor intensive and require a large number of animals due to second-generation assessments (Shinde et al. 2017). At the same time, these animal studies sometimes lack relevance for humans. Human developmental toxicities are evident in only 70–80% of tested rabbits or rodents and the observed responses can be different to those in humans (Olson et al. 2000; Daston and Knudsen 2010). Therefore, in recent years, research has focused on developing alternative human models to animal testing. Human stem cells are a great promise for in vitro test methods for developmental toxicity as they can mimic key aspects of embryonic development (Brickman and Serup 2016; Worley et al. 2018).

One of the most widely used stem cell-based test methods for predicting developmental toxicity relies on the formation of embryoid bodies (EBs) and the differentiation of these into cardiomyocytes. This process mimics the first 3 weeks of human fetal development including the first heart beats on day 21 (Spielmann et al. 1997). Based on this principle, we previously developed the PluriBeat assay with human induced pluripotent stem cells (hiPSC) (Lauschke et al. 2020). The PluriBeat assay employs an 8-day protocol, in which hiPSC are aggregated into EBs and differentiated into beating cardiomyocytes. EBs are exposed to test chemicals for the last 5 days and beating is scored and compared to control conditions (Lauschke et al. 2020). However, scoring of beating EBs is time consuming and labor intensive, as it requires visual inspection of each EB with a light microscope. To conduct future safety assessment of a large number of chemicals as stated in the REACH regulation, assays with increased throughput as well as automated and standardized data acquisition are necessary (Schaafsma et al. 2009; Seiler et al. 2011; Zink et al. 2020).

To improve the readout of our assay, we generated a genetically engineered hiPSC line with a luciferase reporter under control of the cardiac-specific homeobox gene *NKX2.5*. This transcription factor is expressed in cardiac progenitor cells and their progeny and can be detected in mature cardiomyocytes and in the adult mammalian heart (Lints et al. 1993; Lyons et al. 1995; Burridge et al. 2011; Kattman et al. 2011). This makes *NKX2.5* an excellent marker for cardiomyocytes and it has been used in an *NKX2.5-EGFP* human embryonic stem cell (hESC) reporter cell line to monitor cardiac cell populations (Elliott et al. 2011). NKX2.5 is, however, essential for cardiogenesis (Lints et al. 1993), emphasizing the necessity of preserving its functions in a reporter cell line. Using CRISPR/Cas9 technology, we inserted the luciferase reporter immediately downstream of the *NKX2.5* gene with a T2A self-cleaving peptide, ensuring that the NKX2.5 protein is still functional and that the cells can still differentiate into cardiomyocytes. Using this approach, we developed a novel genetically engineered hiPSC-based luciferase reporter gene assay, demonstrated the genetic and functional features of the cells and compared the luminescence intensity response to the beating of EBs upon exposure to test chemicals. We conclude that the PluriLum assay may have the potential to become a valuable new tool for future screening of chemicals for developmental toxicity.

## Materials and methods

### Cell culture

The hiPSC line BIONi010-C was derived from a male donor in the age group 15–19 years with normal disease status and normal karyotype (Bioneer A/S, Hoersholm, Denmark) (Rasmussen et al. 2014). HiPSC were cultured on hESC-Qualified Matrigel-coated (Corning, Corning, USA) cell culture dishes (Thermo Fisher Scientific, Waltham, USA) in mTeSR™1 medium (STEMCELL Technologies, Vancouver, Canada). Medium was exchanged every or every other day and cultures were split approximately once a week using 0.02% EDTA in DPBS and cultured in 5% CO_2_ at 37 °C. Cells were used at passage numbers between 22 and 45. Contamination with mycoplasma was checked regularly using the MycoAlert™ Mycoplasma Detection Kit (Lonza, Basel, Switzerland).

### Genetic engineering

Genetic engineering was performed according to Kim and colleagues, 2014 (Kim et al. 2014). A crispr RNA (crRNA) with the sequence CATGGTATCCGAGCCTGGTA**GGG** (PAM recognition site in bold; Integrated DNA Technologies, Coralville, USA) was designed to target the C-terminal end of the *NKX2.5* gene using a human genomic alignment of *NKX2.5* from https://genome.ucsc.edu/index.html and a CRISPR design tool (https://crispr.cos.uni-heidelberg.de/). The crRNA was annealed to a trans-activating crispr RNA (tracrRNA; Integrated DNA Technologies) to create a single-guide RNA (sgRNA) by mixing and heating to 95 °C followed by cooling to RT on the bench top. A DNA sequence containing the T2A-*Nluc* construct flanked by homologous arms corresponding to 150 bp on each side of the *NKX2.5* STOP codon (Supplementary Material) was acquired from GeneArt (Thermo Fisher Scientific). The plasmid was linearized by restriction enzyme digestion with SphI (New England Biolabs, Ipswich, USA) and purified with a Qiagen Gel extraction kit (Qiagen, Hilden, Germany) according to the manufacturers specifications.

Nucleofection was carried out with a 4D nucleofection device (Lonza, Basel, Switzerland). Briefly, 10 µM of sgRNA and 20 µg of CRISPR-Cas9 nuclease (Integrated DNA Technologies) were mixed and incubated for 15 min at RT to form a ribonucleoprotein (RNP) complex. BIONi010-C wt hiPSC were harvested using Accutase (STEMCELL Technologies) and 1 × 10^6^ cells were transferred to the RNP complex along with 5 µg of the linearized *NKX2.5*-T2A-*Nluc* donor construct. 4D Nucleofection was performed using a P3 Primary cell 4D-nucleofector X Kit L with program CA137 according to the manufacturer’s specifications (Lonza). Two days after nucleofection, gene-edited cells were Accutase treated, passed through a FACS filter and 1 × 10^3^ cells were seeded in a Matrigel-coated 10 cm dish in 5 ml mTeSR1 medium containing 1:10 (v/v) CloneR (STEMCELL Technologies). After 2 days, 5 ml mTeSR1 (1:1) was added, followed by complete medium changes on day 4 and 6. On day 7, single colonies where detached with EDTA and individually transferred to Matrigel-coated 96-well plates in mTeSR1 medium containing 0.1% (v/v) Pen/Strep (Thermo Fischer).

### Genotyping and restriction enzyme digest

After 7-day expansion in 96-well plates, clones were EDTA treated and replica plated to a new Matrigel-coated 96-well dish and a PCR plate for screening of gene-edited clones. The PCR plate was spun down, the supernatant discarded and the cell pellet incubated in 10 µl 1% Qiagen Protease (Qiagen) in H_2_O for 5 min at 75 °C and 5 min at 95 °C and finally diluted 1:10 in H_2_O. PCR with a Exon 2 forward primer and a reverse primer targeting *Nluc* was carried out to reveal colonies where the gene construct had integrated into the genome (Supplementary Table 1). In addition, PCR with primers designed to amplify the entire last part of the *NKX2.5* gene including T2A-*Nluc* was performed. Positive clones were each transferred to two wells of a Matrigel-coated six-well dish and frozen as backup or seeded as single cells for validation of cell clonality.

### Sequencing

Selected clones were analyzed by sequencing of the integration sites. For this, a sequence PCR was performed using a BigDye™ Terminator v3.1 kit (Thermo Fischer) with the Exon 2 forward primer and the 3′UTR reverse primer (Supplementary Table 1). The PCR products were sequenced on a SeqStudio genetic analyzer from Applied Biosciences (Thermo Fischer). The data were analyzed using SnapGene.

### Multi-lineage differentiation potential

Human-induced pluripotent stem cells were Accutase treated (STEMCELL Technologies) at 37 °C for 10 min, counted and centrifuged before being resuspended in mTeSR + 1% RevitaCell.

For ectoderm differentiation, 2 × 10^5^ cells/cm^2^ were seeded per well of a Matrigel-coated 12-well plate. The day after, medium was changed to a 50:50 mixture of DMEM/F12 (Thermo Fisher) and Neurobasel medium (Thermo Fischer) containing 2% (v/v) B27 without vitamin A (Thermo Fisher), 1% (v/v) N2 (Thermo Fisher), 1% (v/v) Glutamax (Thermo Fisher), 0.1% (v/v) Pen/Strep (Thermo Fischer), 10 mM SB431542 (STEMCELL Technologies) and 0.1 μM LDN193189 (STEMCELL Technologies). The medium was exchanged daily for 6 days.

For mesoderm differentiation, 1 × 10^5^ cells/cm^2^ were seeded per well of a Matrigel-coated 12-well plate. The day after, cells were washed in DMEM/F12 (Thermo Fisher) and incubated for 2 days without medium change medium in STEMdiff APEL2 medium (STEMCELL Technologies) containing 25 ng/ml Activin A (Thermo Fischer), 30 ng/ml BMP4 (R&D systems, MN, USA), 50 ng/ml VEGF (Peprotech, Rocky Hill, USA), 1.5 µM CHIR (Axon Medchem, Groningen, Netherlands) and 0.1% (v/v) Pen/Strep (Thermo Fischer). On day 3, the medium was changed to STEMdiff APEL2 medium (STEMCELL Technologies) plus 50 ng/ml VEGF (Peprotech), 10 mM SB431542 (STEMCELL Technologies) and 0.1% (v/v) Pen/Strep (Thermo Fischer) with daily medium changes until day 6.

For endoderm differentiation, 2 × 10^5^ cells/cm^2^ were seeded per well of a Matrigel-coated 12-well plate. The day after, cells were washed once with DDPBS and cultured in MCDC131-1 medium consisting of MCDB131 medium (Thermo Fischer), 5 μg/ml BSA (Biofac, Kastrup, Denmark), 10 mM glucose (Sigma-Aldrich), 1.5 mg/ml NaHCO_3_ (Sigma-Aldrich) and 0.1% Pen/Strep (Lonza), 3 μM CHIR (Selleckchem, TX, USA) and 100 ng/ml Activin A (Cell Guidance systems, Cambridge, UK). On day 2, the medium was changed to MCDC131-1 containing 100 ng/ml Activin A only. The cells were cultured with daily medium changes until day 6.

### Flow cytometry

Gene-edited hiPSC and their in vitro differentiated progeny were detached using Accutase (STEMCELL Technologies), resuspended in 2% BSA (Biofac, Kastrup, Denmark) in DPBS and centrifuged at 300*g* for 5 min. Samples for intracellular markers were fixed and permeabilized using a Foxp3/Transcription factor Staining Buffer set (Thermo Fischer) according to the manufacturer’s specifications and samples for extracellular markers were incubated in 2% BSA (Biofac) in DPBS. 2 × 10^5^ cells pr. sample were transferred to a 96 U-well dish and incubated with fluorescence conjugated antibodies for 1 h at RT (Supplementary Table 2) followed by washing three times in Fix/Perm buffer (intracellular markers) or in 2% BSA (Biofac) in DPBS (intracellular markers). Samples were finally diluted in 2% BSA (Biofac) in DPBS and flow cytometry was performed on a BD Accuri™ C6 Flow Cytometer.

### Immunocytochemistry

The cells were fixed in 4% paraformaldehyde (Merck, Darmstadt, Germany) in DPBS for 10 min, washed in DPBS and permeabilized using 0.5% Triton X100 (Merck, Germany) in 1% BSA (Biofac) for 15 min. The cells were washed in DPBS and blocked using 2% BSA (Biofac) for 1 h followed by incubation with primary antibodies diluted 1:200 in 2% BSA O/N at 4 °C (Supplementary Table 3). The day after, cells were washed three times with DPBS and incubated with secondary antibodies diluted 1:200 in 2% BSA for 1 h at RT (Supplementary Table 3). After a final wash in DPBS, DNA was stained using 5 μg/ml Hoechst (Thermo Fischer). Images were directly acquired on an EVOS FL fluorescence microscope (Thermo Fischer) and processed using Fiji ImageJ software.

### Karyotyping

When cells reached 60–80% confluence they were treated for 1 h with Colcimide (Gibco) followed by Accutase treatment. Single cells were incubated with 0.075 M KCl for 30 min at 37 °C and fixed with 1:3 acidic acid:methanol and sent for G-band karyotyping (University of Tübingen). At least 15 metaphases were counted and 6 of them were structurally evaluated by G-banding and a banding quality of 400–500.

### Cardiomyocyte differentiation

Human-induced pluripotent stem cells were differentiated into cardiomyocytes as described in Lauschke et al. (2020). Essentially, hiPSC were harvested as single cells by incubation in Gibco™ TrypLE™ Select (Thermo Fisher Scientific) for 1–2 min. A single cell suspension of 5 × 10^4^ cells/ml was seeded at 100 µl per well into a 96-well Polystyrene Conical Bottom MicroWell™ Plate (249952, Thermo Fisher Scientific) in mTeSR-ROCK. The plates were centrifuged at 500*g* for 5 min at RT and incubated over night at 37 °C and 5% CO_2_. After 20 h, medium was exchanged by removing 80 µl/well old medium and adding 80 µl/well D0 medium. After this, medium was exchanged daily (24 h ± 2 h) with respective medium on the following days: TS-medium on day 1, Wnt-medium on day 2, TS-medium on day 3 and TS-medium on day 6. 80 µl/well old medium was exchanged for 80 µl/well new medium, except for day 6, where only 60 µl/well were removed. All media components are listed in Supplementary Table 4.

### Luminescence measurement

The EBs were transferred from the conical bottom 96-well plate to a white 96-well plate for luminescence measurements. For this, 150 μl OptiMEM was added per well to dilute the differentiation medium and the EBs transferred in a volume of 50 µl to the white plate using a multichannel pipette. Luminescence was measured using the Promega Nano-Glo Live Cell Assay System (Promega, Madison, USA) according to the manufacturer’s instructions with optimized conditions: Nano-Glo Luciferase Assay Substrate was diluted 1:40 in Nano-Glo Luciferase Assay Buffer. Subsequently, one volume of Opti-MEM I Reduced Serum Medium, no phenol red (Thermo Fisher Scientific) was added, and 25 μl of this diluted substrate added per well of the white plate. The plate was centrifuged briefly and luminescence measurements performed 8 min after substrate addition on a PerkinElmer Enspire 2300 luminometer.

### qRT-PCR analysis

EBs were harvested and RNA extracted with the Qiagen RNeasy Micro Kit (Qiagen) according to the manufacturer’s instructions. The RNA concentration was measured on a Nano Drop (Thermo Fisher Scientific) and 200 µg RNA used for cDNA synthesis using the Omniscript Reverse Transcription Kit (Qiagen). 3.75 ng cDNA was then used per sample for quantitative RT-PCR. This was performed in 384-well plates with technical duplicates for each sample, using the TaqMan Assay Kits listed in Supplementary Table 5 (Thermo Fisher Scientific) and measured on a QuantStudio 7 Flex (Applied Biosystems). Relative gene expression was calculated with the 2^−ΔΔCT^ method relative to the average of the house-keeping genes GAPDH (Glyceraldehyde 3-phosphate dehydrogenase) and ACTB (β-actin). Expression of the house-keeping genes was monitored to be constant. Samples with a cycle threshold (CT) difference > 1 between duplicates were excluded (for samples with CT values < 30 only) and samples with CT values > 35 were regarded as non-detectable.

### Test compound exposure

Thalidomide (T144, Sigma-Aldrich) was prepared as a stock solution in dimethyl sulfoxide (DMSO) at 200 mM and diluted in DMSO 1000 × of the indicated concentrations (0.1, 0.3, 0.6, 1.1, 2.3, 4.5, 9, 18 and 36 µM). The valproic acid (P4543, Sigma-Aldrich) stock solution was prepared in ethanol at 600 mM and diluted in ethanol 1000 × of the test concentrations (25, 50, 100, 200 and 300 µM). We showed previously that both compounds are not cytotoxic at these concentrations in our cells (Lauschke et al. 2020).

For exposure during cardiomyocyte differentiation, the diluted stocks were added 1:1000 to the respective media on day 1, 2, 3 and 6. DMSO/ethanol concentrations were kept constant across all EBs and control EBs were exposed to DMSO or ethanol 1:1000, respectively. For each concentration, 32 EBs were exposed. On day 7, the beat score was assessed and luminescence measured. The beat score was assessed by evaluating the beating of the EBs visually using a light microscope for up to 10 s with the following criteria: if the entire EB was contracting, a beat sore of 2 was given. If only parts of the EB were moving, a beat score of 1 was given, and if there was no movement visible in the EB, a beat score of 0 was given. We found it important to discriminate between these categories, because a crude categorization into beating and no beating would fail to account for subtle effects.

### NanoLuc inhibition study

Cells on day 7 of the assay were exposed for 1 h with 36 µM thalidomide, as this was not expected to affect cardiomyocyte differentiation and contraction but at the same time would allow for direct inhibition of the NanoLuc enzyme within the cells. For this, cells were differentiated without exposure for 7 days. On the last day, medium was exchanged to TS containing 36 µM thalidomide or DMSO control (32 EBs each), and the cells incubated for 1 h. The beat score was assessed before and after medium change and 1-h incubation to be the same. Thereafter, luminescence was measured.

### Data processing

All experiments were performed in biological triplicates with 32 EBs per condition. The beat score was calculated as the average of 32 EBs in each experiment.

Statistical analysis was performed in GraphPad prism version 8. For the luminescence time course, the average luminescence intensity of 32 wells was calculated and the average values of three biological experiments analyzed. First, normality tests were performed (Anderson–Darling, D’Agostino-Pearson omnibus, Shapiro–Wilk and Kolmogorov–Smirnov). Because the data were not normally distributed, a Kruskal–Wallis test and multiple comparisons with Dunn’s correction for comparison to day 0 were performed. To analyze whether gene expression differed significantly between cell lines, a two-way ANOVA without matching was performed.

Luminescence intensity measurements upon test compound exposure were analyzed according to the following procedure: first, values from wells without EBs were removed (Luminescence value < 50,000). Then, outliers from 32 EBs per concentration were removed (approximately 3%) by deleting values that differed more than three standard deviations from the average. For calculating the normalized luminescence intensity, the average of the controls was calculated and the average of each concentration normalized to that average. For statistical analysis, the normalized luminescence intensity for each concentration was analyzed in GraphPad prism version 8 with a one-way ANOVA without matching followed by multiple comparisons using Dunnett’s correction, where each compound concentration was compared to the control.

The beat score for the high test concentrations of thalidomide (shown in Fig. 3) was analyzed in R Studio. An ordinal logistic regression was performed using the polr package with beat score and concentration set as factors. The analysis was based on > 90 EBs from three independent experiments.

Absolute IC_50_ values were calculated in GraphPad prism (version 8) with a four-parameter logistic curve fit with the lower limit set to 0 and the upper limit to 1, as well as the parameter F set to 50 to get the absolute inhibition of 50%. Calculations were based on the normalized luminescence intensity of > 80 EBs from three independent experiments per cell line.

Stars on all figures indicate the following significance: *p < 0.05, **p < 0.01, ***p < 0.001.

## Results

### Creating the *NKX2.5-T2A-Nluc* cell line

**Fig. 1:**
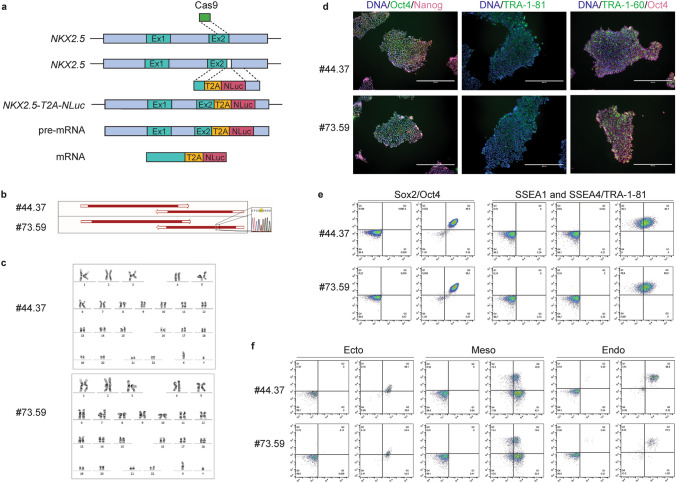
Genetic engineering and validation of cell lines. **a** Schematic presentation of genetic engineering strategy. **b** Sequencing results of mutated areas in #44.37 and #73.59. **c** Karyotyping of #44.37 and #73.59. **d** Immunofluorescence staining of pluripotency markers in #44.37 and #73.59, scale bar = 400 µm. **e** Flow cytometry analysis of pluripotency markers, left panels show controls, right panels the respective antibody combinations: Sox2/Oct4, SSEA1 and SSEA4/TRA-1-81. **f** Flow cytometry analysis of differentiation into derivatives of the three germ layers: Ecto- (Pax6/Sox1), Meso- (CD34/CD56) and Endoderm (CD184/Sox17)

We set out to create the *NKX2.5-T2A-Nluc* reporter cell line based on the hiPSC line BIONi010-C according to the gene-editing strategy depicted in Fig. 1a. Screening and sequencing of the potential clones revealed that two clones had both alleles with *NKX2.5-T2A-Nluc* in frame and both of them were homozygous (BIONi010-C-*NKX2.5-T2A-Nluc*-44.37 (#44.37) and BIONi010-C-*NKX2.5-T2A-Nluc*-73.59 (#73.59). While clone #44.37 had the DNA construct perfectly incorporated on both alleles, clone #73.59 had a point mutation (G → T) on one allele in the 3′UTR downstream of the coding region of *Nluc* (Fig. 1b). As the mutation was not part of the coding region of *NKX2.5* or *Nluc*, it was not seen as a basis for discarding the clone.

The two clones were expanded and subjected to detailed quality control assessments to verify their pluripotency and karyotypes. First of all, we assessed the karyotype which was normal in both cell lines (Fig. 1c). We also analyzed the expression of pluripotency markers in the two clonal cell lines by immunocytochemistry. As shown in Fig. 1d, both lines expressed Oct3/4, Nanog, TRA-1-81, TRA-1-60 and Oct4, suggesting that the cells retained their pluripotency during the genetic engineering. To get a quantitative measure of the proportion of pluripotent cells in the cell populations, we performed flow cytometry with pluripotency markers. We found that both clonal cell lines were > 99% double positive for Sox2 and Oct4, > 99% positive for SSEA4 and less than 1% SSEA1 positive (Fig. 1e). Only approximately 60% of the cells were TRA-1-81 positive but this is in line with previous observed staining results of hiPSC lines in our laboratory and, therefore, potentially due to the antibody used. To confirm the ability of the cells to form derivatives of the three germ layers, we subjected them to differentiation protocols into ectoderm, mesoderm and endoderm. Flow cytometry analysis of the populations showed that a high proportion of the cells expressed markers for the respective germ layers (Fig. 1f). Together with qualitative analysis of cell morphologies, this indicated that the cells had indeed differentiated into derivatives of the three germ layers. In conclusion, we have proven that the two clonal cell lines BIONi010-C *NKX2.5-T2A-Nluc*-44.37 and BIONi010-C-*NKX2.5-T2A-Nluc*-73.59 retained their pluripotency, so we continued to characterize them for use in our developmental toxicity assay.

### Cardiomyocyte differentiation of the reporter cell lines

**Fig. 2:**
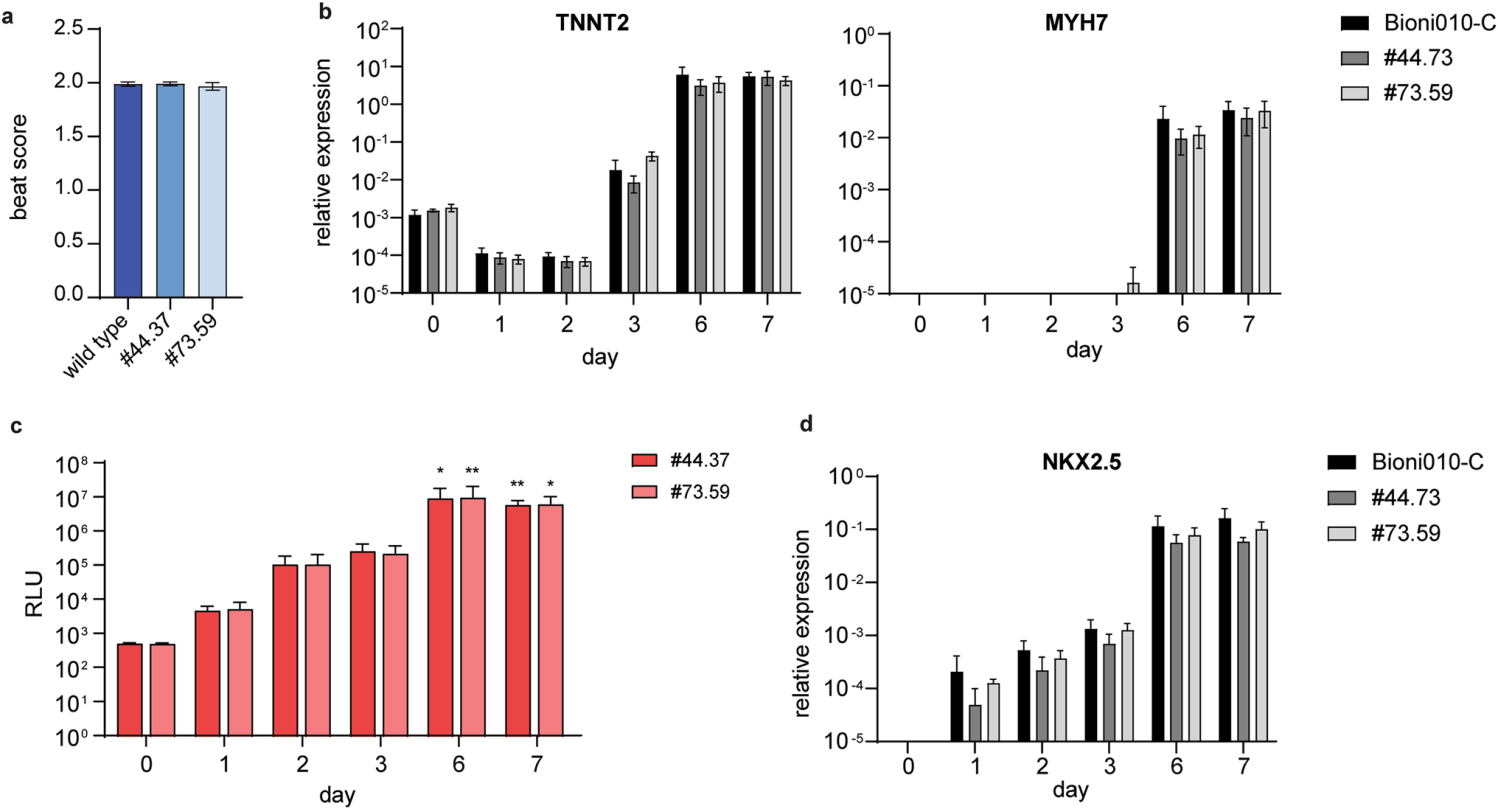
Differentiation into cardiomyocytes. **a** Beat score of EBs on day 7 of differentiation into cardiomyocytes for BIONi010-C wild type, clone #44.37 and #73.59. **b** Expression of cardiac marker genes TNNT2 and MYH7 for clone #44.37 and #73.59 during the course of differentiation. **c** Luminescence intensity during the course of differentiation into cardiomyocytes of clone #44.37 and #73.59. **d** Expression of NKX2.5 for clone #44.37 and #73.59 during the course of differentiation. Mean and SD (**a**, **c**) or mean and SEM (**b**, **d**) of 32 EBs in *n* = 3 experiments

First of all, it was important to prove that the differentiation into cardiomyocytes was not hampered by the genetic engineering. To this end, we differentiated the two reporter cell lines and the wild-type cell line BIONi010-C in parallel. All three cell lines gave rise to a beat score of almost 2, showing that all EBs were contracting on the last day of differentiation (Fig. 2a). This indicated that the differentiation efficiency was the same in the reporter clones and the wild-type cell line. To also investigate this at the molecular level, we analyzed the expression of cardiomyocyte specific genes in the differentiated cells from all three cell lines. We detected *TNNT2* in pluripotent hiPSC on day − 1 and decreasing on day 0 and 1, before it was upregulated by five orders of magnitude on day 6 and 7. *MYH7* was only detectable on day 6 and 7. Both markers indicated the presence of cardiomyocytes on day 6 and 7 (Fig. 2b). Importantly, there was no difference between expression levels in the three cell lines, which was confirmed by a two-way ANOVA analysis. Having established that there was no difference in cardiomyocyte differentiation between the two reporter cell lines and the wild type, we measured luminescence intensity during the course of differentiation. We found a strong and significant increase over time in both clones of the reporter cell line (Fig. 2c) while we did not detect any luminescence signal above background for the wild type (data not shown). This correlated well with increased expression of *NKX2.5* during the course of differentiation (Fig. 2d). Moreover, *NKX2.5* was expressed at similar levels in the two cell lines, which we confirmed by a two-way ANOVA analysis. Together, these data strongly indicated that luminescence intensity truly mirrored expression of *NKX2.5* and was indicative of cardiomyocyte differentiation.

### Luminescence intensity as a readout for developmental toxicity

**Fig. 3:**
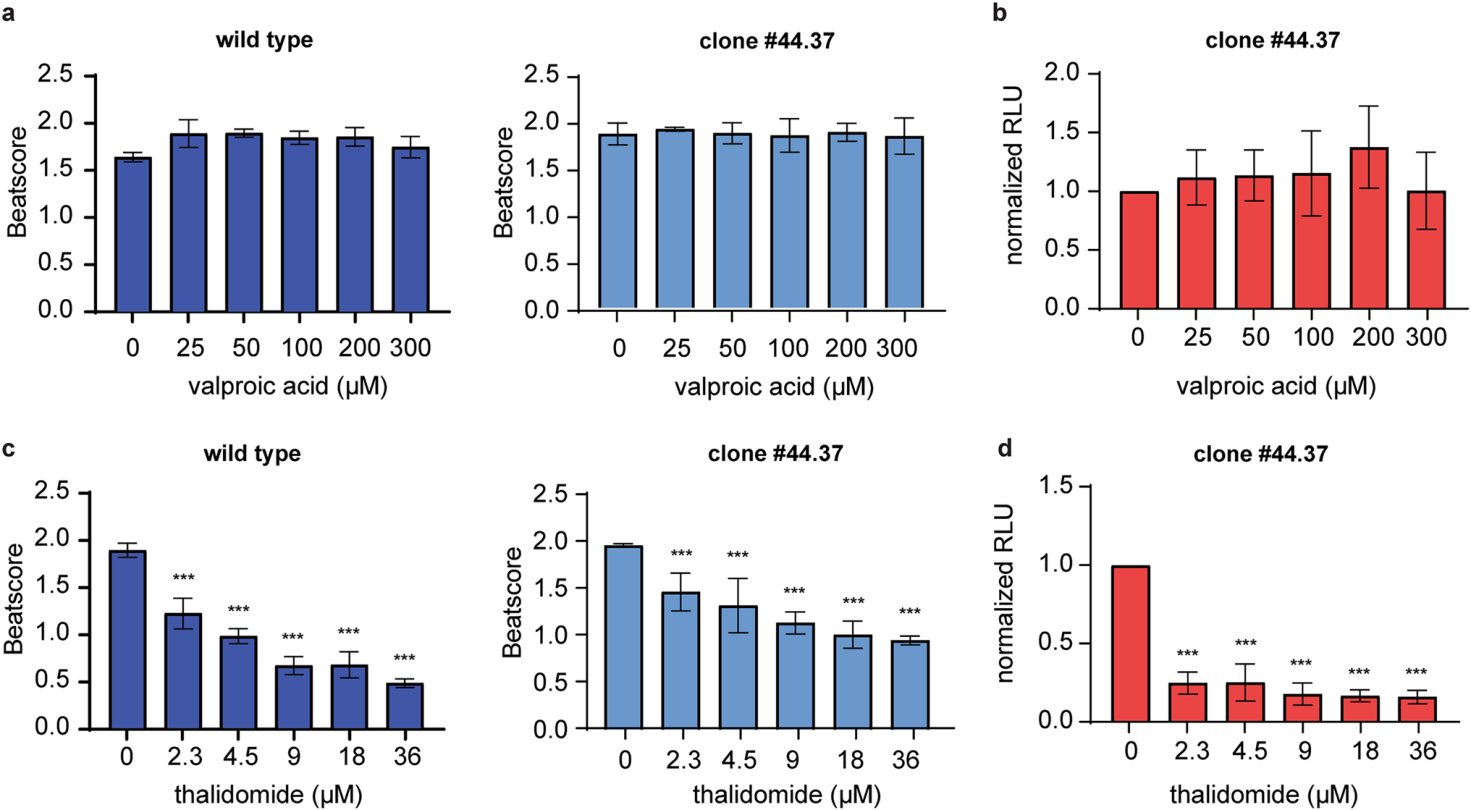
Testing the reporter cell line for use as a developmental toxicity assay. **a** Valproic acid exposure of BIONi010-C wild-type and clone #44.37. **b** Luminescence intensity in clone #44.37. **c** Thalidomide exposure of BIONi010-C wild type and clone #44.37. **d** Luminescence intensity in clone #44.37. Mean and SD of 32 EBs in n = 3 experiments

**Fig. 4:**
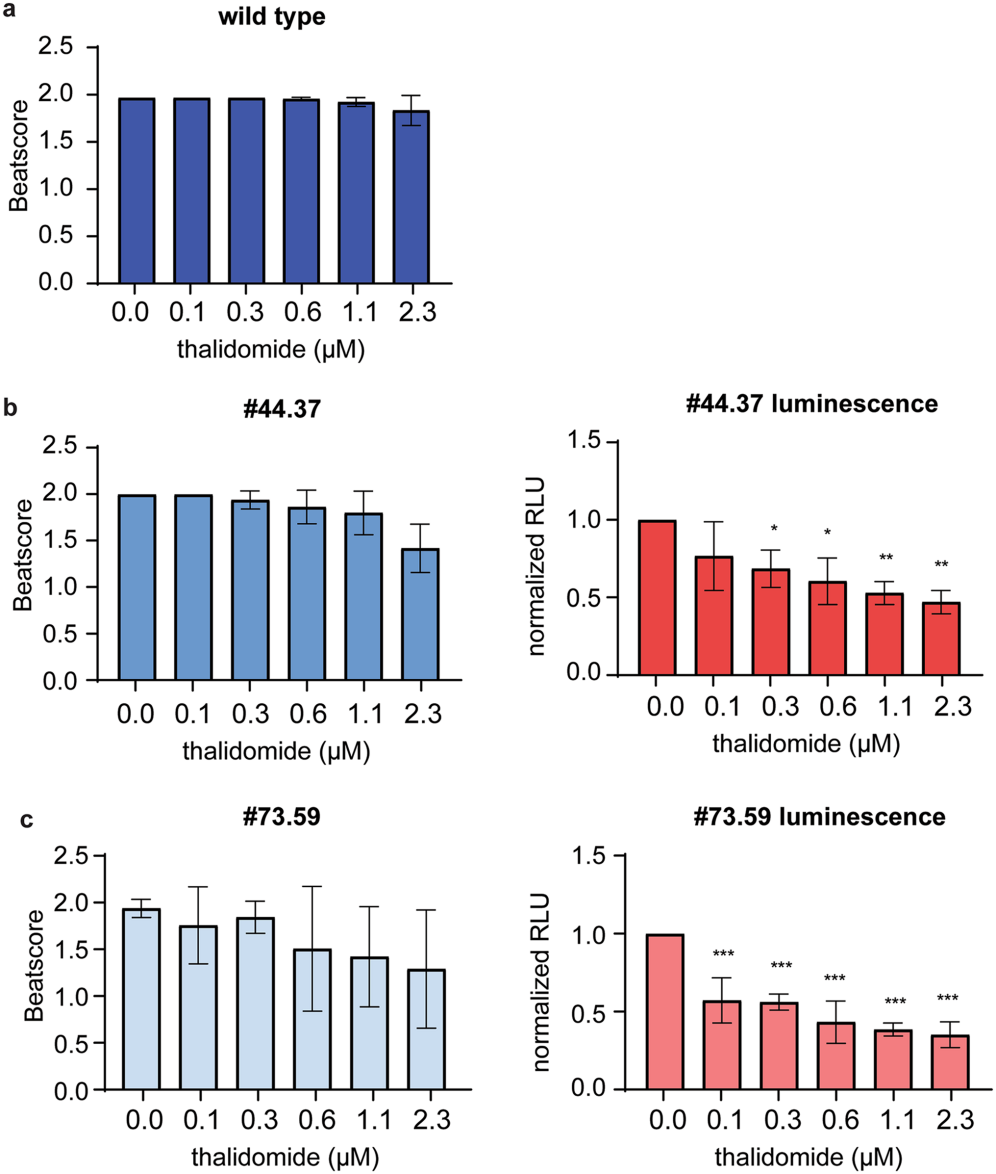
Luminescence intensity is a more sensitive readout than the beat score. **a** Exposure with lower concentrations of thalidomide, beat score of BIONi010-C wild type. **b** Beat score and luminescence intensity in clone #44.37. **c** Beat score and luminescence intensity in clone #73.59. All experiments were performed in biological triplicates with *n* = 32 EBs in each replicate. Mean and SD of 32 EBs in *n* = 3 experiments

After having established that the two clonal reporter cell lines differentiated similarly into cardiomyocytes as the original wild-type BIONi010-C cell line, we tested the performance of luminescence intensity as a readout for developmental toxicity. Because there was no apparent difference between the two clones, we did not find it necessary to test both identical clonal cell lines and, therefore, chose to continue with only one of them, clone #44.37. We selected two chemicals that we had previously tested in the PluriBeat assay, namely valproic acid and thalidomide. Valproic acid is negative in the PluriBeat assay, whereas thalidomide is positive with an IC_25_ of 2.0 µM (Lauschke et al. 2020). As expected, valproic acid did not decrease the beat score in BIONi010-C wild type and the #44.37 clone at the tested concentrations (Fig. 3a). The luminescence intensity was not decreased either (Fig. 3b), illustrating that luminescence measurements did not give rise to false positive results compared to beating cardiomyocytes in this case. Thalidomide exposure led to a concentration-dependent decline in the beat score in BIONi010-C wild type and #44.37 (Fig. 3c). Intriguingly, the decrease in luminescence intensity in #44.37 was observed at much lower concentrations than that of the beat score, and maximum efficacy was reached already at the lowest thalidomide concentration tested (Fig. 3d). Therefore, we reduced the thalidomide concentrations to find the linear range of the luminescence response. At these lower concentrations, we found no significant decreases of the beat score in either BIONi010-C (Fig. 4a) or #44.37 (Fig. 4b). Contrary, the luminescence intensity in #44.37 was significantly decreased from 0.3 µM thalidomide (LOEC value in Table 1) and showed a clear concentration-dependent decline (Fig. 4b). To confirm that the assay was that sensitive for thalidomide, we tested the low concentrations also in clone #73.59. This confirmed our findings, as the luminescence intensity was decreased significantly already at 0.1 µM (Table 1, Fig. 4c). For comparisons with our previously published data on thalidomide toxicity, we calculated the absolute IC_50_ values based on luminescence intensity upon exposure to the lower thalidomide concentrations. As presented in Table 1, we found an IC_50_ of 1.55 µM in clone #44.37 and 0.35 µM in #73.59 (for curve fitting see Suppl. Fig. 1B).

**Table 1:** LOEC (lowest observed effect concentration) and absolute IC_50_ values for thalidomide tested in the two clonal cell lines with luminescence intensity as a readout

Cell line	LOEC (µM)	IC_50_ (µM)
BIONi010-C-*NKX2.5-T2A-Nluc*-44.37	0.3	1.55 (1.10–2.59)
BIONi010-C-*NKX2.5-T2A-Nluc*-73.59	0.1	0.35 (0.25–0.46)

To exclude that the marked response in the luciferase assay was due to inhibition of NanoLuc by thalidomide, we performed a direct enzyme inhibition study. After short-term exposure with thalidomide on day 7 of the assay, the luminescence readout was not affected (Suppl. Fig. 1). This suggests that the decrease in luminescence intensity upon exposure to thalidomide was not due to a direct inhibition of the NanoLuc enzyme. Therefore, we conclude that luminescence intensity is a more sensitive readout for developmental toxicity of thalidomide than the beat score in our assay.

## Discussion

Here, we generated a luciferase-*NKX2.5* reporter gene assay based on hiPSC and a luminescence readout which we termed the PluriLum assay. To our knowledge, this is the first engineered hiPSC reporter line with a potential use for testing chemicals for developmental toxicity in vitro.

We chose to link luciferase expression to the cardiac marker gene *NKX2.5*, which is expressed during cardiomyocyte differentiation from the cardiac progenitor stage through to cardiomyocytes (Burridge et al. 2011; Kattman et al. 2011). Our gene expression data confirmed this and showed that *NKX2.5* expression and luminescence intensity increased continuously from days 1 to 7 in the same pattern. Because *NKX2.5* is essential for cardiomyocyte differentiation, we had to design a gene-editing strategy that did not interfere with protein function. Therefore, we targeted the luciferase gene downstream of the *NKX2.5* coding region and separated the two sequences by a T2A signal to get two separate proteins synthesized. We showed that NKX2.5 function was not inhibited because cardiomyocyte differentiation was as efficient in the two reporter cell lines as in the wild type. To our knowledge, this is the first reporter cell line with a functional *NKX2.5* gene, whereas the widely used *NKX2.5-EGFP* hESC line carries only one wild-type allele and one disrupted *NKX2.5* allele (Elliott et al. 2011). Furthermore, we decided to use luminescence rather than fluorescence intensity as a readout, because it has a lower background light intensity, which leads to a higher relative signal and, therefore, higher assay sensitivity (Fan and Wood 2007). Our data on thalidomide support this notion and indicated that our luminescence-based assay may have a high sensitivity and improved assay performance. Other reporter cell lines for developmental toxicity testing based on a fluorescence readout or β-galactosidase staining did not show increased sensitivity compared to the conventional readout of beating cardiomyocytes (Kugler et al. 2015, 2016). Furthermore, luminescence assays are less susceptible to autofluorescent chemicals than fluorescence-based assays (Fan and Wood 2007). This is an advantage when screening large sets of chemicals as testing for autofluorescence can be omitted. However, chemicals can inhibit luciferase in rare cases (Thorne et al. 2012; Ho et al. 2013; Walker et al. 2017) and we, therefore, showed that thalidomide does not inhibit NanoLuc directly.

The two clonal cell lines #44.37 and #73.59 differentiated with the same efficiency into cardiomyocytes, however, they showed minor differences in sensitivity towards exposure to thalidomide. While there was almost no effect on the beat score in #44.37 at concentrations below 2.3 µM thalidomide, there seemed to be a decreasing trend in #73.59. This was also reflected by the decrease of luminescence intensities, which were less pronounced in #44.37 than in #73.59. These deviations are deemed within the normal variation range that can often be observed between cell experiments.

Both clonal cell lines gave LOEC and IC_50_ values in the PluriLum assay that are significantly lower than our previously published values on thalidomide in the PluriBeat assay. Here, we found IC_50_ values ranging from 0.3 to 1.55 µM, while we previously reported an IC_25_ value of 2.0 µM in BIONi010-C wild type based on the beat score. Of note, we were not earlier able to calculate the IC_50_ on beat score as the efficacy on the beat score was not that strong and instead the IC_25_ was calculated (Lauschke et al. 2020). The sensitivity of the PluriLum assay for thalidomide seems to be similar to or lower than potency values reported previously in other test systems: thalidomide has been reported to give IC_50_ values of 0.5 µM (Kameoka et al. 2014), approximately 1 µM (Palmer et al. 2013), 38 µM (Mayshar et al. 2011) and 450 µM (Aikawa et al. 2014). Thus, the PluriLum assay can detect developmental toxicity of thalidomide with either a better or a similar sensitivity compared to other assays. Importantly, it is more sensitive than the other two assays based on EB differentiation (Mayshar et al. 2011; Aikawa et al. 2014) as well as our own published PluriBeat assay (Lauschke et al. 2020). Other reporter cell line assays have not reported on thalidomide toxicity (Uibel et al. 2010), potentially, because these studies were mostly based on mouse ESCs. Thalidomide has not been detected in mouse-based assays which might be because the teratogenic effects of thalidomide are not observed in mice (Schumacher et al. 1970; Vargesson 2015).

Contrary, valproic acid was negative in our PluriLum assay, as it was in the PluriBeat assay. Even if the clinical use of valproic acid has been associated with congenital malformations of newborns (Tomson et al. 2011), valproic acid is often negative in many in vitro assays based on cardiomyocyte differentiation and has mostly been detected in assays based on general developmental or neurodevelopmental mechanisms (Uibel et al. 2010; Palmer et al. 2013; Kameoka et al. 2014; Shinde et al. 2017). Thus, we hypothesize that PluriLum may not detect neurodevelopmental toxicants, but this has to be tested in future experiments. Of note, we did not find any difference in the effects of valproic acid on the readouts of beating cardiomyocytes and luminescence, indicating that both readouts detect the same mechanisms of developmental toxicity.

In our PluriLum assay, we have not normalized the luminescence intensity to protein content or cell number, because we found that the total cell number of each EB was most likely an integral part and indicator of our luminescence readout. We have shown previously that EB size decreases upon exposure to thalidomide but not valproic acid (Lauschke et al. 2020). The total luminescence intensity of each EB decreases with reduced size and contributes to the high sensitivity of the assay. We, therefore, hypothesize that a reduction in luminescence intensity captures two mechanisms of action, namely a chemical’s effect on proliferation (reduced EB size) as well as on differentiation (reduced *NKX2.5-Nluc* expression). However, this has to be addressed more closely in future studies.

In conclusion, we provide a proof-of-concept that a genetically engineered hiPSC line with luciferase in the locus of *NKX2.5* could be developed and used in an assay that we have termed the PluriLum assay. We validated pluripotency and genetic stability of the clonal reporter cell lines and proved their capacity to differentiate into cardiomyocytes. We showed that luminescence intensity is a sensitive readout for the teratogenic effect of thalidomide, which has not been reported for other reporter cell lines yet. Valproic acid was negative in the PluriLum assay as it was shown earlier in the PluriBeat assay. Our conclusion is that we have developed a new assay that seems promising as a tool for screening of chemicals for developmental toxicity. Testing of more chemicals in the future is needed for a more detailed characterization of the assay and to path the way toward a wider usage of the PluriLum assay.

## Supplementary Information

Below is the link to the electronic supplementary material.

Supplementary file1 (DOCX 143 KB)
