# Supplementary material for: Creating a human-induced pluripotent stem cell-based NKX2.5 reporter gene assay for developmental toxicity testing
Source: Arch Toxicol. 2021 Mar 4;95(5):1659–70. doi: 10.1007/s00204-021-03018-y (PMC8113199; doi:10.1007/s00204-021-03018-y)
Supplement: Supplementary file 1 — Supplementary file1 (DOCX 143 KB) [file 204_2021_3018_MOESM1_ESM.docx]

**Supplementary material**

**Creating a human-induced pluripotent stem cell-based NKX2.5 reporter gene assay for developmental toxicity testing**

Karin Lauschke^1,2,^*, Andreas Frederik Treschow^1,3,^*, Mikkel Aabech Rasmussen^3^, Nichlas Davidsen^1^, Bjørn Holst^3^, Jenny Emnéus^2^, Camilla Taxvig^1^ and Anne Marie Vinggaard^1^

*1: National Food Institute, Technical University of Denmark, Kongens Lyngby, Denmark*

*2: Department for Biotechnology and Biomedicine, Technical University of Denmark, Kongens Lyngby, Denmark*

*3: Bioneer A/S, Hørsholm, Denmark*

** authors contributed equally*

**Sequence of the T2A-Nluc construct targeting the NKX2.5 coding region**

CCCGCCGGGCCTTCCCCAGCGCAGCCGGCCACTGCCGCCGCCAACAACAACTTCGTGAACTTCGGCGTCGGGGACTTGAATGCGGTTCAGAGCCCCGGGATTCCGCAGAGCAACTCGGGAGTGTCCACGCTGCATGGTATCCGAGCCTGGGGCAGTGGAGAGGGCAGAGGAAGTCTGCTAACATGCGGTGACGTCGAGGAGAATCCTGGCCCAATGGTGTTCACCCTGGAAGATTTCGTCGGCGACTGGCGGCAGACAGCCGGCTATAATCTGGACCAGGTGCTGGAACAAGGCGGCGTGTCCAGCCTGTTTCAGAACCTGGGAGTGTCTGTGACCCCTATCCAGCGGATTGTGCTGAGCGGCGAGAACGGCCTGAAGATCGACATCCACGTGATCATCCCTTACGAGGGCCTGTCCGGCGATCAGATGGGACAGATCGAGAAGATCTTTAAGGTGGTGTACCCCGTGGACGACCACCACTTCAAAGTGATCCTGCACTACGGCACCCTGGTCATCGATGGCGTGACCCCAAACATGATCGACTACTTCGGCAGACCCTACGAGGGAATCGCCGTGTTCGACGGCAAGAAAATCACCGTGACCGGCACACTGTGGAACGGCAACAAGATCATCGACGAGCGGCTGATCAACCCCGATGGCAGCCTGCTGTTCAGAGTGACCATCAACGGCGTGACAGGATGGCGGCTGTGCGAGAGAATTCTGGCCTGAGGAAGGGACCCGCGTGGCGCGACCCTGACCGATCCCACCTCAACAGCTCCCTGACTCTCGGGGGGAGAAGGGGCTCCCAACATGACCCTGAGTCCCCTGGATTTTGCATTCACTCCTGCGGAGACCTAGGAACTTTTTCTGTCCCACGCG

Exon 2 overhang, T2A sequence, Nluc, 3’UTR overhang

**Supplementary table 1**: PCR primers for screening clones:

| **Primer** | **Sequence (5’-3’)** |
| --- | --- |
| NKX2.5 Exon 2 Fwd | TATAACGCCTACCCCGCCTA |
| Nluc Rv | CCGACGAAATCTTCCAGGGT |
| NKX2.5 3’UTR Rv | CAGGCTGCAGGATCACTCA |

**Supplementary table 2**: Antibodies for flow cytometry:

|  | **Antibody** |
| --- | --- |
| **Pluripotency** | OCT4 PE (BD, 560186)  SOX2 Alexa Fluor 647 (BD, 560294)  SSEA4 PE (BD, 560128)  TRA-1-81 Alexa Flour 647 (BD, 560793)  SSEA1 PE (BD, 560142) |
| **Ectoderm** | PAX6 Alexa 647 (BD, 562249)  SOX1 PE (BD, 561592) |
| **Mesoderm** | CD34 PE (BD, 555822)  CD56 APC (BD, 555518) |
| **Endoderm** | CD184 PE (BD, 555974)  SOX17 Alexa 647 (BD, 562594) |

**Supplementary table 3**: Antibodies for immunocytochemistry:

| **Primary antibodies** | **Secondary antibodies** |
| --- | --- |
| Mouse anti OCT3/4 (STEMCELL Technologies, 01550)  Rabbit anti NANOG (Sigma-Aldrich, AB5731) | Alexa Flour 488 Donkey anti-mouse IgG (Thermo Fisher, A-21202)  Alexa Flour 594 Donkey anti-Rabbit IgG (Thermo Fisher, A-21207) |
| Mouse anti TRA-181 (BioLegend, 330702) | Alexa Flour 488 Donkey anti-mouse IgG (Thermo Fisher, A-21202) |
| Goat anti OCT4 (Abcam, AB27985)  Mouse anti TRA-1-60 (Merck, MAB4360) | Alexa Fluor 594 Donkey anti-Goat IgG (Thermo Fisher, A-11058)  Alexa Flour 488 Donkey anti-mouse IgG (Thermo Fisher, A-21202) |

**Supplementary table 4**: Media for cardiomyocyte differentiation:

| **mTeSR-ROCK** | |
| --- | --- |
| mTeSR1 (STEMCELL Technologies 85850) | 10 ml |
| ROCK (5 mM, Cayman Chemical 10005583) | 20 µl |
| PSG (Life technologies 10378016) | 100 µl |
| **DO medium** | |
| KO-DMEM | 10 ml |
| PSG (Life technologies 10378016) | 100 μl |
| ITS (BD 354351) | 10 μl |
| Y (10 mM, Abcam 120129) | 10 μl |
| FGF2 (10 μg/ml, Peprotech 100-18B) | 10 μl |
| Activin A (10 μg/ml, eBioscience 34-8993-85) | 10 μl |
| CHIR (1 mM, Axon Medchem 1386) | 25 μl |
| BMP4 (10 μg/ml, R&D 314-BP-010) | 1 μl |
| **TS medium** | |
| KO-DMEM (Life technologies 10829018) | 10 ml |
| PSG (Life technologies 10378016) | 100 μl |
| TS * | 100 μl |
| Asc (250 mM, Sigma-Aldrich 49752) | 10 µl |
| **Wnt medium** | |
| KO-DMEM (Life technologies 10829018) | 10 ml |
| PSG (Life technologies 10378016) | 100 μl |
| TS * | 100 μl |
| Asc (250 mM, Sigma-Aldrich 49752) | 10 μl |
| Wnt (2mM, Tocris Cat.-No. 5148) | 20 μl |

*TS is prepared by adding 1ml of sodium selenite (27 mg sodium selenite Sigma-Aldrich S5261 in 400 ml DPBS w/o Ca and Mg) to the 99 ml transferrin (55 mg transferrin Sigma-Aldrich T8158 in 99 ml DPBS w/o Ca and Mg)

**Supplementary table 5:** TaqMan Assays for quantitative RT-PCR:

| **Gene** | **TaqMan Assay** |
| --- | --- |
| *GAPDH* | Hs02786624_g1 |
| *ACTB* | Hs01060665_g1 |
| *NKX2.5* | Hs00231763_m1 |
| *MYH7* | Hs01110632_m1 |
| *TNNT2* | Hs00943911_m1 |


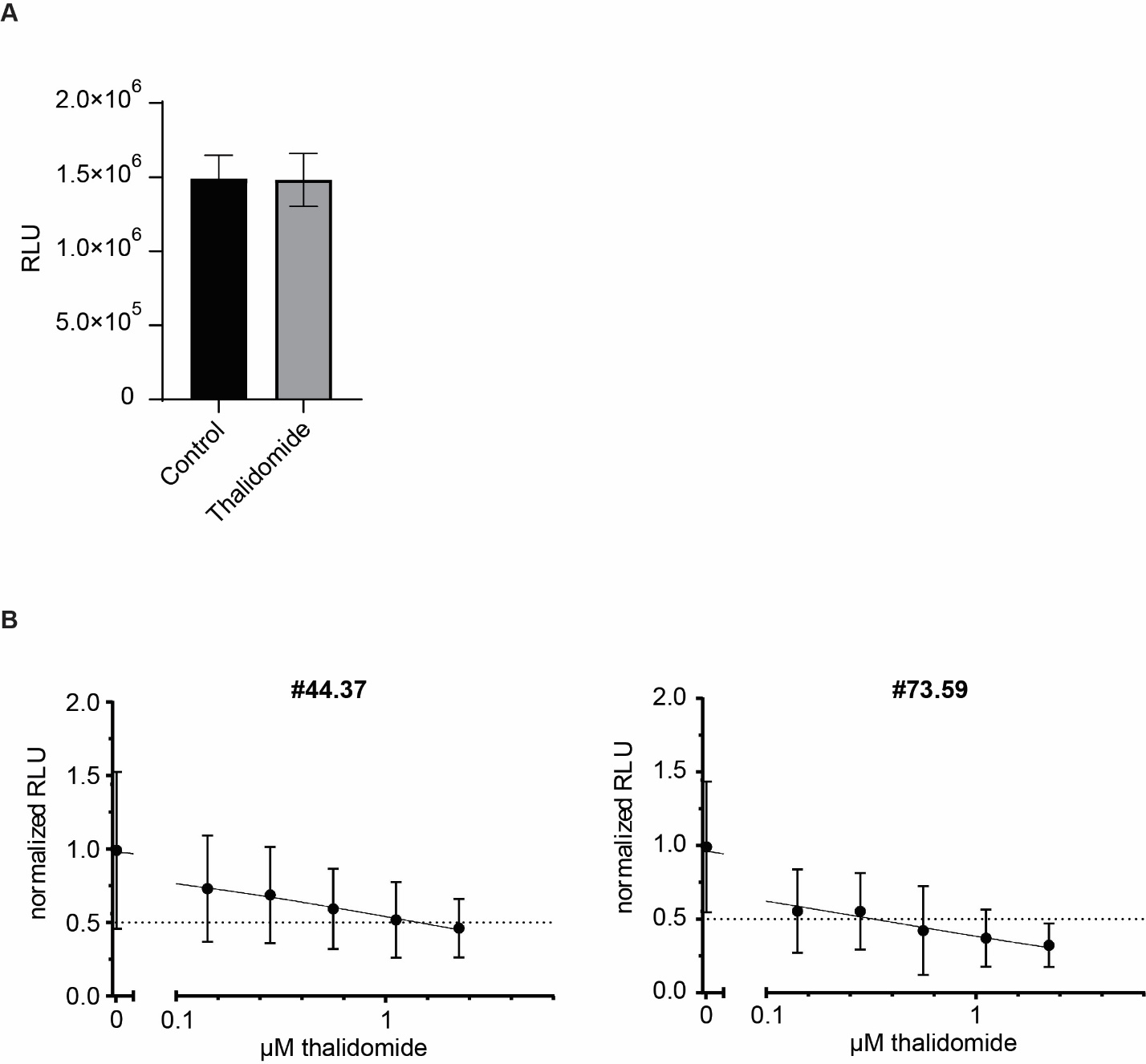


**Supplementary Fig. 1: A)** Direct inhibition of NanoLuc. Differentiated cells on day 7 were exposed for one hour to thalidomide and luminescence measured. Mean ± SEM of 32 EBs. **B)** Non-linear regression curves for calculations of IC_50_ values. Mean and SD of three independent experiments per cell line.
